# PDMS as a Substrate for Lipid Bilayers

**DOI:** 10.1021/acs.langmuir.3c00944

**Published:** 2023-07-26

**Authors:** James A. Goodchild, Danielle L. Walsh, Harrison Laurent, Simon D. Connell

**Affiliations:** † Molecular and Nanoscale Physics Group, School of Physics and Astronomy, University of Leeds, Leeds LS2 9JT, United Kingdom; ‡ Bragg Centre for Materials Research, William Henry Bragg Building, University of Leeds, Leeds LS2 9JT, United Kingdom

## Abstract

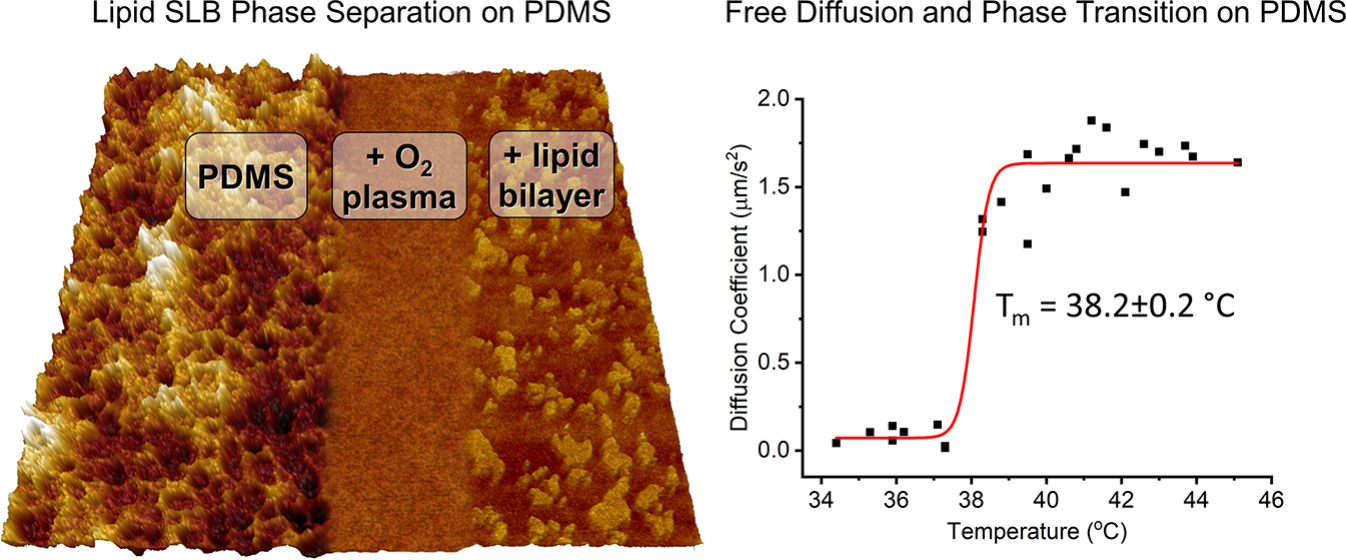

PDMS (polydimethylsiloxane) is a cheap, optically clear polymer that is elastic and can be easily and quickly fabricated into a wide array of microscale and nanoscale architectures, making it a versatile substrate for biophysical experiments on cell membranes. It is easy to imagine many new experiments will be devised that require a bilayer to be placed upon a substrate that is flexible or easily cast into a desired geometry, such as in lab-on-a-chip, organ-on-chip, and microfluidic applications, or for building accurate membrane models that replicate the surface structure and elasticity of the cytoskeleton. However, PDMS has its limitations, and the extent to which the behavior of membranes is affected on PDMS has not been fully explored. We use AFM and fluorescence optical microscopy to investigate the use of PDMS as a substrate for the formation and study of supported lipid bilayers (SLBs). Lipid bilayers form on plasma-treated PDMS and show free diffusion and normal phase transitions, confirming its suitability as a model bilayer substrate. However, lipid-phase separation on PDMS is severely restricted due to the pinning of domains to surface roughness, resulting in the cessation of lateral hydrodynamic flow. We show the high-resolution porous structure of PDMS and the extreme smoothing effect of oxygen plasma treatment used to hydrophilize the surface, but this is not flat enough to allow domain formation. We also observe bilayer degradation over hour timescales, which correlates with the known hydrophobic recovery of PDMS, and establish a critical water contact angle of 30°, above which bilayers degrade or not form at all. Care must be taken as incomplete surface oxidation and hydrophobic recovery result in optically invisible membrane disruption, which will also be transparent to fluorescence microscopy and lipid diffusion measurements in the early stages.

## Introduction

Supported lipid bilayers (SLBs) are a powerful tool for investigating many membrane phenomena such as phase separation, molecular diffusion, and lipid ordering.^1−4^ They are experimentally accessible to numerous surface-sensitive techniques such as atomic force microscopy (AFM),^5,6^ quantitative crystal microbalance with dissipation (QCM-D),^7,8^ and fluorescence recovery after photobleaching (FRAP)^9,10^ and can be used in biotechnological applications such as pharmaceutical or protein biosensor assays.^11^ The most common substrates for SLBs include glass, mica, and silicon, the choice dictated by the choice of technique.

PDMS (polydimethylsiloxane) is a cheap, optically clear polymer that is easy and fast to fabricate into a wide array of microscale and nanoscale geometries. This versatility can be exploited to explore many different membrane properties and phenomena. Hovis and Boxer first demonstrated lipid self-assembly on PDMS, with hydrophobic PDMS surfaces supporting monolayers and plasma-oxidized hydrophilic PDMS surfaces supporting bilayers.^12,13^ They showed how PDMS can be used for patterning bilayer arrays in chosen geometries using oxidized PDMS stamps.^12,13^

Forming SLBs on deformable PDMS enables the observation of membrane buckling and deformation from protein assemblies^14^ as well as the study of T-cell activation on substrates with physiological levels of mechanical resistance.^15^ Topographically patterned PDMS with controllable curvature allows selective localization and study of biological components such as phase-separated domains and proteins, which align with specific areas of bilayer curvature.^16−19^ PDMS can also be cast against microspheres or nanoparticles to form substrates for microcavity-supported bilayers in which the bilayer is suspended across a void and proteins can be studied in a freely diffusing environment without substrate interactions.^20,21^ The direct integration of bilayer membranes with PDMS microfluidic devices enables rapid, low-concentration, and high-throughput immunoassays^22,23^ and also helps facilitate further advances in biological on-chip applications such as organ-on-chip.

There are many good experimental reasons for using PDMS as a substrate for SLB’s, and there is no doubt that new experiments will be devised that require a bilayer to be placed upon a substrate that is either flexible or easily cast into a desired geometry. However, PDMS also has its drawbacks and limitations, and the extent to which the behavior of the membrane is affected on PDMS has not been fully explored. In this paper, we use AFM and fluorescence optical microscopy to investigate the use of PDMS as a substrate for the formation and study of SLBs. Bilayers are formed on PDMS slabs, and the effect of PDMS on the dynamics and transition temperatures of the lipids are measured. Previously, lipid phase separation has only been observed under limited conditions, either in surface-fused, immobile GUV patches that replicate the pre-existing GUV domain structure^24^ or in more mobile GUV patches ruptured on top of a pre-formed lipid layer.^17^ We show that lipid phase separation does occur directly on plasma-treated PDMS supported bilayers in a bilayer with molecular diffusion identical to glass and very similar to mica. Despite this molecular mobility, there is a severe restriction in domain growth caused by the nanoscale surface roughness. We show high-resolution AFM images of the porous structure of pristine PDMS and the extreme smoothing effect of oxygen plasma treatment used to hydrophilize the surface. Despite this decrease in roughness, it is not sufficient to allow domain formation. Once formed, the bilayer structure will degrade over hour timescales correlated with the previously well characterized hydrophobic recovery of PDMS. The critical degree of hydrophobicity for bilayer breakdown is indicated by a water-in-air contact angle of approximately 30° achieved after a few hours in water-aged PDMS. In summary, we explore the behavior of PDMS-supported lipid bilayers and compare to other commonly used bilayer substrates, glass and mica, how this might lead to different interpretation of experimental data,^25^ informing future experiments of lipid membranes on flexible PDMS.

## Materials and Methods

### Preparation of the Lipid Vesicles

DOPC (1,2-dioleoyl-*sn*-glycero-3-phosphocholine), DPPC (1,2-dipalmitoyl-*sn*-glycero-3-phosphocholine), and 16:0 NBD PE [1,2-dipalmitoyl-*sn*-glycero-3-phosphoethanolamine-*N*-(7-nitro-2-1,3-benzoxadiazol-4-yl) (ammonium salt)] were purchased from Avanti Polar Lipids (Alabaster, AL). Texas Red DHPE (Texas Red 1,2-dihexadecanoyl-*sn*-glycero-3-phosphoethanolamine, triethylammonium salt) was purchased from Thermo Fisher Scientific UK. Vesicles were prepared as described previously.^25^ Each lipid was dissolved into an individual 5 mM CHCl_3_ stock solution, mixed in the desired composition, dried under nitrogen, and vacuum-desiccated overnight. The dry film was hydrated in ultrapure water (Milli-Q) to 1 mg/mL, vortexed for 30 min, heated in an oven at 50 °C for 30 min, and then tip-sonicated for 30 min at 4 °C to form small unilamellar vesicles (SUVs). The SUV sample was centrifuged at 3000 rpm for 3 min to remove metal sonicator tip sediment from the SUVs.

### Preparation of PDMS

PDMS base and Sylgard 184 cross-linker (Dow Corning) were mixed in a 10:1 ratio and stirred thoroughly for 2 min, degassed by centrifuging at 4000 rpm for 1 min, and vacuum-desiccated for 15 min. For contact angle measurements, the degassed mixture was cured into slabs in a plastic Petri dish at 70 °C for 30 min, cut into 1 cm^2^ pieces, and glued with epoxy to glass microscope slides. PDMS substrates for AFM and fluorescence spectroscopy were prepared by spin-coating (Laurel Technologies, WS-640 MZ) a small drop of the degassed PDMS mixture on a glass cover slip at 1700 rpm for 60 s accelerating at 200 rpm/s and then curing it on a hot plate at 95 °C for 10 min. PDMS was oxidized using a Diener Electronic Zepto Oxygen Plasma Laboratory Unit for 2 min at 0.3–0.4 mBar (100 W, 40 kHz). Oxidized PDMS for forming SLBs was used immediately.

### Supported Bilayer Formation

Details of substrate preparation and bilayer formation on mica (Agar Scientific) and glass (Thermo Scientific, Menzel-Glaser) are described previously.^25^ Bilayer formation on PDMS is similar. For fluorescence measurements, oxidized spin-coated PDMS on glass cover slips were assembled into a home-built flow cell consisting of a sealed incubation chamber around the substrate and an inlet and outlet for flowing the sample in and washing. One milliliter of 1 mg/mL lipid vesicles were injected into the cell and incubated on the surface for 30 min at room temperature for DOPC and at 50 °C for DPPC and DPPC/DOPC). One milliliter of 20 mM MgCl_2_ at the same temperature was added and incubated for a further 30 min. The sample was then allowed to cool to room temperature, and washed to remove any unfused vesicles by flowing room-temperature MiliQ water through the cell at 1 mL/min for 30 min. For AFM measurements, 100 μL of SUV solution was deposited onto an oxidized spin-coated PDMS sample on a glass cover slip and incubated in a sealed humidity chamber for 1 h at 50 °C. Halfway through incubation, 100 μL of 20 mM MgCl_2_ was added. After incubation, the bilayer was cooled to room temperature and rinsed to remove any unruptured vesicles by pipetting 50 μL of MilliQ water across the surface 10 times.

To measure the bilayer temperature, a thermocouple was positioned in the buffer close to the substrates in the fluorescence fluid cell and in the AFM incubation dish, as described previously.^25^ The cooling rate was determined using a temperature range to match the transition temperature of the system (DPPC/DOPC (60:40), 33–29 °C).^25−27^ Two different cooling rates were achieved by removing samples from the oven (0.25 °C/min) or by turning the oven off (slow cooled 0.08 °C/min).

### Contact Angle

Static contact angle measurements (described previously^25^) were taken using a First Ten Angstroms FTA 4000 CAG. A droplet of MilliQ water, approximately 0.2 μL, was pipetted onto the surface, and an image was captured. Contact angle measurements were taken at specific time points after plasma oxidation. The nominal instrumental uncertainty is ±2°, but replicate measurements were more reproducible.

### Fluorescence and Fluorescence Recovery after Photobleaching

Fluorescence microscopy was performed, as described previously,^25^ using a Nikon Eclipse E600 microscope with an Andor Technology Zyla cCMOS camera. The microscope was equipped with a mercury lamp and filter cubes suitable for Texas Red (ex = 540–580, em = 600–660) and NBD (ex = 465–495, em = 515–555) and ×40 air and ×100 oil objectives.

Briefly, an aperture was used to photobleach a 30 μm diameter circular bilayer area for 30 s, and then the recovery of fluorescence due to diffusing lipid molecules was imaged at 3 s intervals for 3 min (3 s lag between bleaching and first measurement). The fluorescence intensity value in the image stack was normalized by the analysis macro, which set the bleached spot intensity in the first image recorded at 3 s to zero and the unbleached background fluorescence to 1.0. The exponential recovery is fitted to obtain a characteristic recovery half-life (), which can then be converted to a diffusion coefficient (*D*).
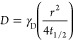
where *r* is the radius of the bleach spot and γ_D_ is a constant (0.88) related to the circular bleach shape. Diffusion coefficient values on PDMS are averages of four repeat runs; for each repeat run, at least five different areas from the substrate were imaged. Independent-sample *t* tests were performed using IBM SPSS Software.

### Thermal Transition Temperature of the Supported Lipid Bilayers

**Figure 1:**
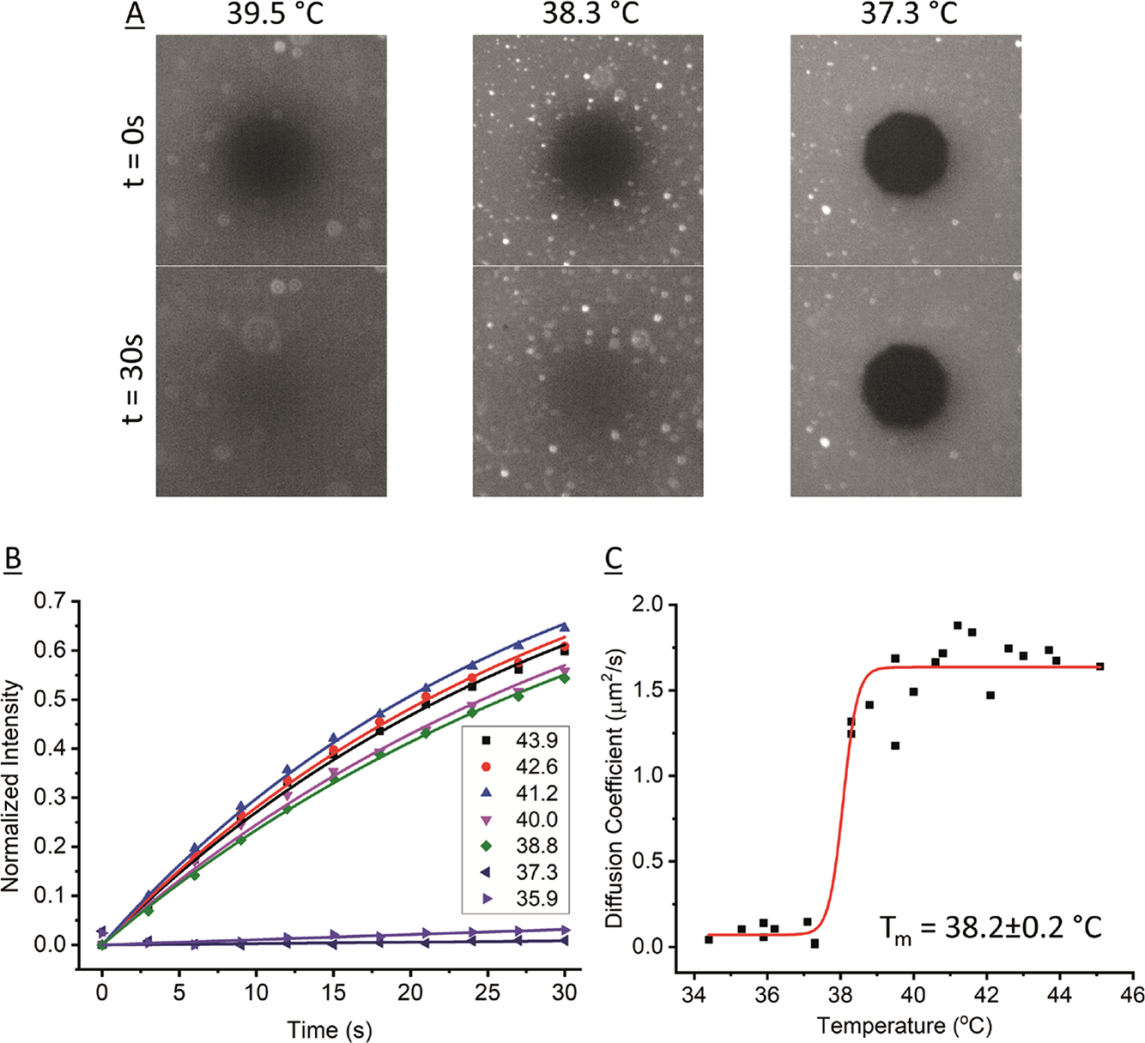
FRAP on DPPC + 0.5 mol % NBD bilayer on PDMS as the bilayer cools. (A) Fluorescence images at different timepoints after photobleach as the bilayer cools at selected temperatures. Diameter of circular bleach area is 30 μm. (B) Example fluorescence recovery curves (normalized intensity) versus time at different temperatures with exponential recovery fits. (C) Calculated diffusion coefficients at each temperature; data taken from three repeat experiments overlaid on the same axes. Data fitted to a Boltzmann sigmoid, *T*_m_ = 38.2 ± 0.2 °C.

The bilayer in the flow cell was heated to 60 °C, and FRAP images were taken during passive and continuous cooling to ambient temperature. FRAP recovery slows dramatically at the thermal transition, *T*_m_, as the bilayer converts from the liquid phase to the solid phase (Figure 1). With a cooling rate of 0.6 °C min^–1^ in the range of 45–35 °C, the time to capture fluorescence recovery was limited to 30s for each data point to maintain temperature precision (i.e., a 0.3 °C change). To determine precise diffusion coefficients, a full recovery to a stable value (normalized intensity of 1) should be captured (Figure 1B), but this was not necessary to determine an accurate *T*_m_, the purpose of this experiment. Nevertheless, a clear recovery curve was measured, and the diffusion coefficient was reported here. Diffusion coefficient vs temperature plots were fitted to a Boltzmann sigmoidal curve.
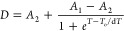
where *A*_1_ and *A*_2_ represent the approximately steady diffusion coefficients above and below the thermal transition and *T*_o_ is the midpoint of the curve, which is taken as the value of *T*_m_.

The FRAP data was collected during cooling and thus compared to cooling-scan DSC data in this paper. Heating and cooling rates can offset *T*_m_ values slightly. The cooling rate for pure DPPC samples during FRAP in the fluorescence fluid cell (0.6 °C/min) was calculated between 45 and 35 °C to match the DSC *T*_m_ of pure DPPC (40.73 ± 0.03 °C).^25^ This is different to the cooling rate calculated for domain cooling of DPPC:DOPC (60:40) under the same ambient conditions (0.25 ± 0.02 °C/min), which was calculated at a different temperature range (33–29 °C) to match the *T*_m_ of DPPC:DOPC (60:40). For a detailed discussion regarding how cooling rates in FRAP and DSC affect the *T*_m_, see our previous paper and its Supporting Information.^25^

### Atomic Force Microscopy

AFM images were acquired using a Bruker Dimension AFM with an ICON head with ScanAsyst Fluid probes (0.7 N/m, 150 kHz, Bruker Probes) in liquid peak force tapping mode. This mode was used to allow direct control of the imaging force at 150–300 pN to minimize differential compression of different lipid phases. The image pixel resolution was 768 pixels × 768 pixels minimum. The AFM *z*-noise floor is 0.03 nm (as shown on atomically smooth mica after cleavage; Figure S2). The ultimate *xy* lateral resolution of the AFM is dependent on the tip sharpness, image size, and pixel rate. The smallest lipid domains we detect are approximately 5 nm in diameter, giving us an estimate of lateral resolution.

### Image Analysis

All image analysis procedures are similar to methods described previously.^25^ Fluorescence microscopy images were analyzed and processed using the FIJI distribution of ImageJ (NIH). AFM images were analyzed using Nanoscope Analysis v1.9 (Bruker). AFM images were flattened using the appropriate order of leveling with thresholding for each image. Throughout this paper, uncertainties are quoted as the larger value between the standard error and the instrument uncertainty.

*R*_a_ roughness was measured using a built-in Nanoscope Analysis function. *R*_a_ values were measured over either 1 or 5 μm^2^ images (stated). For the porous polymer structure in pre-plasma-treated PDMS (Figure 6A), the images were acquired at the smaller image size of 1 μm^2^ with a higher pixel rate and AFM tracking needed to resolve the structure. These were directly compared to post-plasma-treated PDMS images of the same size (Figure 6B). After plasma treatment, a larger image of the smoother structure was acquired to enable direct comparison with glass and mica (Figure S2). Roughness values generally increase with the size of the image because larger-length scale fluctuations dominate smaller fluctuations, e.g., post-plasma PDMS gives 0.28 ± 0.03 nm (5 μm^2^) and 0.21 ± 0.03 nm (1 μm^2^) over repeat images. This highlights the limitations of using *R*_a_ roughness measurements alone, even with the same image size. The effect of the roughness can be dependent on its spatial frequency as well as amplitude, which is essential when trying to link substrate topography to bilayer behavior. For this reason, power spectra (Figure 6D) were measured using Nanoscope analysis, giving a quantitative roughness (power of height fluctuations) vs wavelength. Pore size and depth were also measured using the Nanoscope Analysis section function.

Domains are often non-circular, so diameters were estimated by fitting an ellipse to the domain using Particle Analysis in ImageJ and then taking the average of the long and short axes. The radially averaged correlation function was calculated from AFM images flattened in Nanoscope and converted to a binary image using thresholding in ImageJ. Autocorrelation plots were generated using a radially averaged autocorrelation function macro^28^ and fitted to an exponential decay to give a characteristic correlation length (Figure S5).
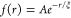
where *f*(*r*) is the autocorrelation function, *r* is the distance, and ξ is the correlation length. This correlation length method was used for domains on PDMS, which due to their complex morphologies could not be fit individually to calculate a domain size. More details on correlation length analysis of domain structure can be found in our previous publication.^25^

## Results

### Molecular Diffusion on PDMS

PDMS has been used as a substrate for cell mimetic systems in multiple studies, with bilayers formed on oxidized hydrophillic PDMS showing free diffusion.^12,23,29−32^ To validate this behavior in our system and allow direct comparison with other surfaces, we formed DOPC + 0.5 mol % TR-DHPE bilayers on spin-coated, oxidized PDMS and confirmed by FRAP that the lipids freely diffuse, *D* = 1.04 ± 0.03 μm^2^/s (Figure S6). This is on the low side of the 1–2 μm^2^/s range found in the literature, but more importantly, there was no significant difference to the diffusion we found on glass, *D* = 1.02 ± 0.04 μm^2^/s (*t*(14) = 0.20, *p* = 0.84), or on mica, *D* = 0.96 ± 0.04 μm^2^/s (*t*(8) = 1.32, *p* = 0.42), using the same instrumentation and protocol as determined in previous work in our lab.^25^ In comparison, Faysal et al.^30^ also found the diffusion coefficient on oxidized PDMS (*D* = 1.42 ± 0.03 μm^2^/s) to be similar to glass (*D* = 1.39 ± 0.05 μm^2^/s). On PDMS, we also observe free diffusion in DPPC + 0.5 mol % NBD at 45 °C (in the fluid phase above *T*_m_) , with *D* = 1.6 ± 0.2 μm^2^/s (Figure 1). This is a drop of 24% compared to DPPC on mica (2.1 ± 0.1 μm^2^/s) and glass (2.1 ± 0.03 μm^2^/s) under the same conditions, as also found in our previous work.^25^ This experimental variability is not unusual. When fluid-phase lipid diffusion on PDMS is compared directly to glass in the literature, it is shown to be faster,^12^ similar,^30^ or 50% slower.^23^ Diffusion on PDMS is clearly variable when compared to glass, and there are currently no other direct comparisons of diffusion between diffusion on PDMS vs mica. This variability can be attributed to the wide range of experimental parameters when hydrophilizing the PDMS with plasma or UV irradiation. For example, Zhao et al. comprehensively and systematically varied the plasma power and duration as well as the subsequent storage conditions in air and in different liquids, tracking the changes in contact angle vs time with somewhat varying results.^33,50^ Often, no explicable trend was found.

Importantly, lipids form bilayers that can freely diffuse on PDMS, and thus, it can be used as a substrate for model systems, but to check its validity as a substrate for forming biologically relevant bilayers, we tested other characteristic properties of the supported lipid bilayers: liquid–solid transition temperature, the ability to phase separate and form domain structures, and long term stability.

### Transition Temperature on PDMS

FRAP-with-temperature was used to measure the transition temperature (*T*_m_) of lipids on PDMS. When the DPPC bilayer is cooled and passes through *T*_m_, it changes from a liquid phase to a gel phase and the photobleached area will stop recovering (Figure 1).^1,25,34^ The *T*_m_ is marginally lower on PDMS, 38.2 ± 0.2 °C, than for free-floating multilamellar vesicles (MLVs) measured by differential scanning calorimetry (DSC) in our previous study, 39.73 ± 0.02 °C.^25^ It is also lower than on mica, 40.2 ± 0.3 °C, but similar to glass, 38.6 ± 0.2 °C, both measured using the exact same protocol as our previously published work.^25^ We attributed this drop in *T*_m_ on glass compared to mica to the increased roughness of the surface, disrupting the lipid packing.^25^ Confirming that the bilayer can go through phase transitions on PDMS is further evidence that PDMS substrates enable good model bilayer systems and that the substrate does not influence lipid order in the bilayer very much. To our knowledge, this is the first time phase transitions have been measured on PDMS.

### Phase Separation on PDMS

The phase separation of lipid species has been extensively studied in model systems, such as GUVs (giant unilamellar vesicles), GPMVs (giant plasma membrane vesicles), and SLBs on mica, as the “lipid raft” theory implicates these phases in biological processes such as cell signaling and protein accumulation.^1,5,35,36^ As phase separation is a characteristic property of bilayers arising from multiple factors, such as difference in lipid order, line tension, and bilayer fluidity, we tested a well-known mixture that easily exhibits solid–liquid phase separation on PDMS.

**Figure 2:**
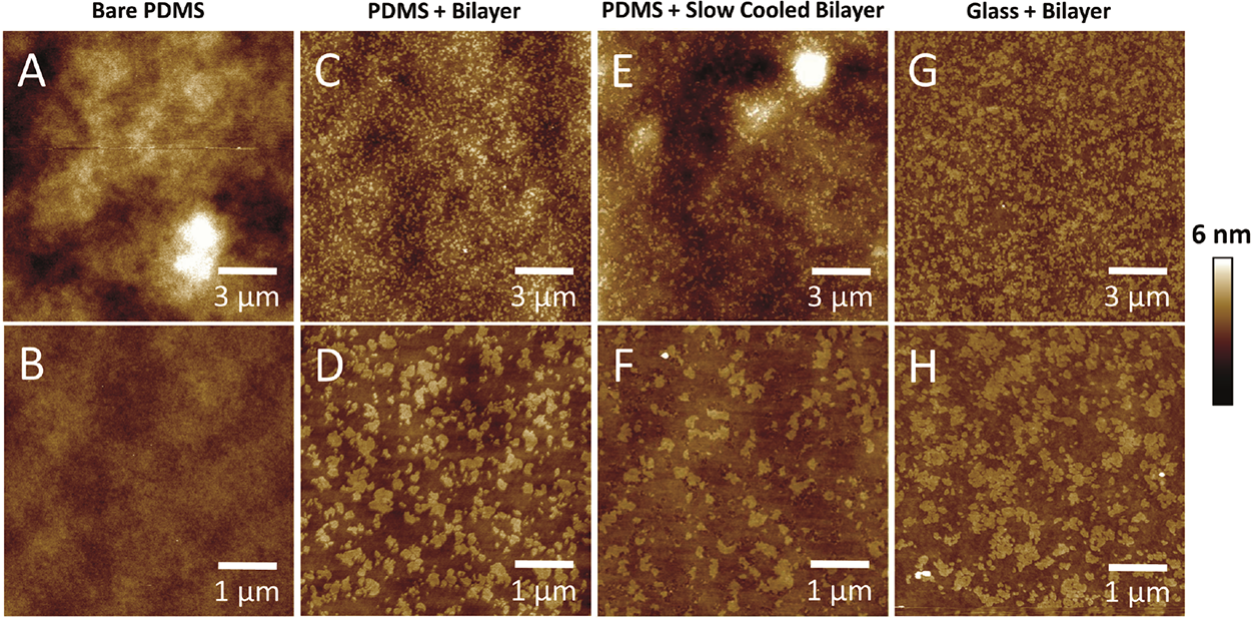
AFM imaging used to discriminate sub-optical resolution domains. (A, B) AFM images of plasma-oxidized PDMS with no bilayer. (C, D) AFM images of plasma-oxidized PDMS with a DPPC/DOPC (60:40) bilayer showing nanoscale domains. (E, F) Here, the bilayer has been cooled slowly in an attempt to grow the size of the nucleated domains to little effect. (G, H) AFM images of piranha- and UV ozone-cleaned glass with a DPPC/DOPC (60:40) lipid bilayer showing nanoscale domains comparable to those on PDMS. All bilayers were cooled from an incubation temperature of 50 °C to room temperature at 0.25 ± 0.02 °C/min except for the slow-cooled bilayer on PDMS at 0.080 ± 0.008 °C/min. Due to the slow cool, the time between the plasma treatment of PDMS and imaging is longer, and therefore, areas of instability in the bilayer can be seen as PDMS starts to hydrophobically recover (more information later in the hydrophobic recovery section).

**Figure 3:**
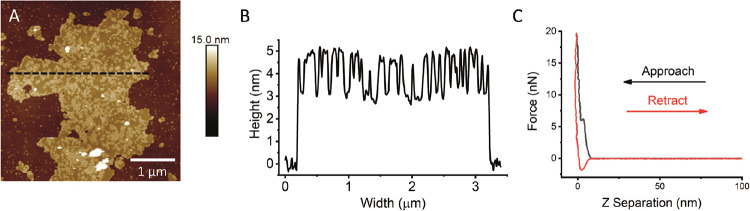
(A) Patch of DPPC/DOPC (60:40) bilayer on PDMS showing phase separation. (B) Height profile of the white line in (A) showing the height of the bilayer from the PDMS substrate and the height of the gel and fluid phases. (C) Example of a force curve on a DPPC/DOPC (60:40) bilayer on PDMS showing the characteristic bilayer rupture. The compliance of the PDMS can also be observed in the non-linearity of the retract curve.

**Figure 4:**
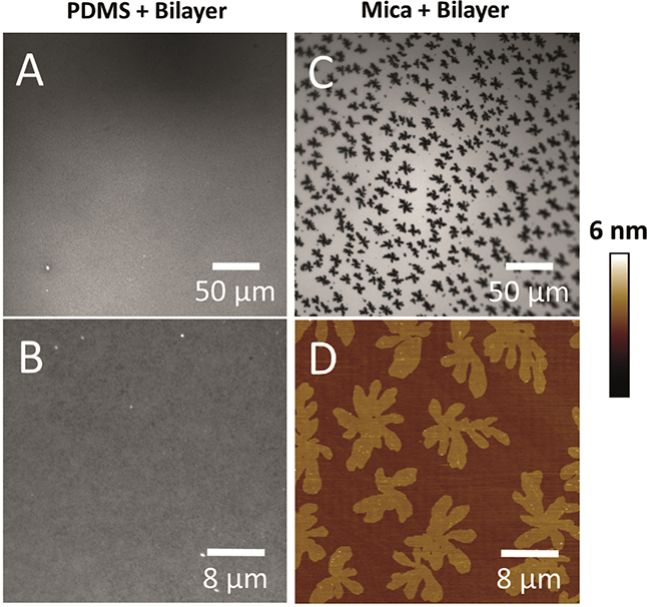
(A, B) Fluorescence microscopy images of plasma-oxidized PDMS with a DPPC/DOPC (60:40) + 0.5 mol % TR-DHPE bilayer showing an apparently homogenous bilayer, although some indistinct contrast can be detected at a higher magnification (B). (C, D) Same lipid composition on mica showing large, well-defined domains up to 10 μm in size. (C) is a fluorescence image at the same magnification as (A). (D) is an AFM image at the same magnification as (B).

DPPC/DOPC (60:40) bilayers were prepared on plasma-oxidized PDMS, and nanoscale phase separation was observed between the gel and liquid phases using AFM (Figure 2C,D). Although we strive to produce defect-free bilayers, sometimes, incomplete bilayer formation can be helpful in showing isolated patches of bilayer on PDMS, allowing accurate measurement of bilayer depth. Figure 3A shows such a patch of the same lipid mixture, unambiguously confirming the presence of a bilayer with a height of 5 nm and a 1.5–2.0 nm height difference between the gel and fluid phases (Figure 3B). Force spectroscopy also confirmed the presence of a bilayer due to the characteristic rupture curve at 5–6 nN and depth of (again) 5 nm (Figure 3C).^37,38^ The uniformity of the domain structure reveals that the influence of the exposed bilayer edge does not extend a long distance into the patch, although gel phases do tend to form at the periphery. These nanoscale domains on PDMS were not observed when imaged using fluorescent microscopy because they are below the diffraction limit (Figure 4A,B), although a speckle pattern at the limit of resolution can be seen similar to that observed on glass,^25^ showing that there is some structure. This explains why phase-separated lipid domains are not observed optically on PDMS. The nanoscale domains on PDMS are in stark contrast to the micrometer-scale domains formed on mica (Figure 4) (and commonly observed in GUVs^39^) but are similar to the domains observed on glass (Figure 2), all using the same lipid mixture and incubation conditions.^25^ As well as solid–liquid systems, Honigmann et al. show that the size of lipid domains in the liquid–liquid phase separating systems are also hindered on glass compared to mica.^40^

Due to the small, irregular, and partially connected domains on PDMS, particle analysis methods failed to accurately characterize the size distribution of the domains, so correlation length was used instead. The correlation length gives a quantitative measure of domain size and distribution based on the radially averaged distance between two different sets of binary pixels in a binarized domain image (Figure S5).^25,41,42^ The domains on PDMS (49 ± 7 nm) are around 2 orders of magnitude smaller than the domains on mica (2.3 ± 0.4 μm) and the same order of magnitude as domains on glass (74 ± 5 nm)(Table S1).^25^

### Domain Size Changes with Cooling Rate

In an attempt to create phase-separated domains on a larger scale, the cooling rate of the deposited bilayer was slowed down. Briefly, according to standard nucleation theory, slowing the rate gives more time for the diffusing molecules to reach the nucleating domain and reduces the probability of super-saturation, leading to fewer but larger domains. The characteristic size of the domains on mica increases by 43% as the cooling rate is slowed from 0.25 ± 0.02 to 0.080 ± 0.008 °C/min, as expected (Table S1).^25^ As these domains on mica are large and well-separated, they could be thresholded and measured by more straightforward particle analysis software. Fitting domains to ellipses showed a similar 55% increase in domain size with decreased cooling rate, showing that the correlation length is a reliable measure of domain size.^25^ It should be noted that correlation length underestimates the size of these large solid-phase domains due to the fractal like protrusions on mica, which will have more non-domain pixels closer to the center of the domain compared to the edge of a fitted ellipse. For further detail, refer to our previous paper and its SI.^25^ However, on PDMS, slowing the cooling rate did not increase domain size, indicating that the usual mechanism of domain growth has been arrested or prevented from taking place (Figure 2 E + F). Surprisingly, the slow-cooled domains were slightly smaller at 37 ± 8 nm compared to 49 ± 7 nm for the ambiently cooled domains on PDMS.

### Hydrophobic Recovery of PDMS and Effect on Bilayer Stability

**Figure 5:**
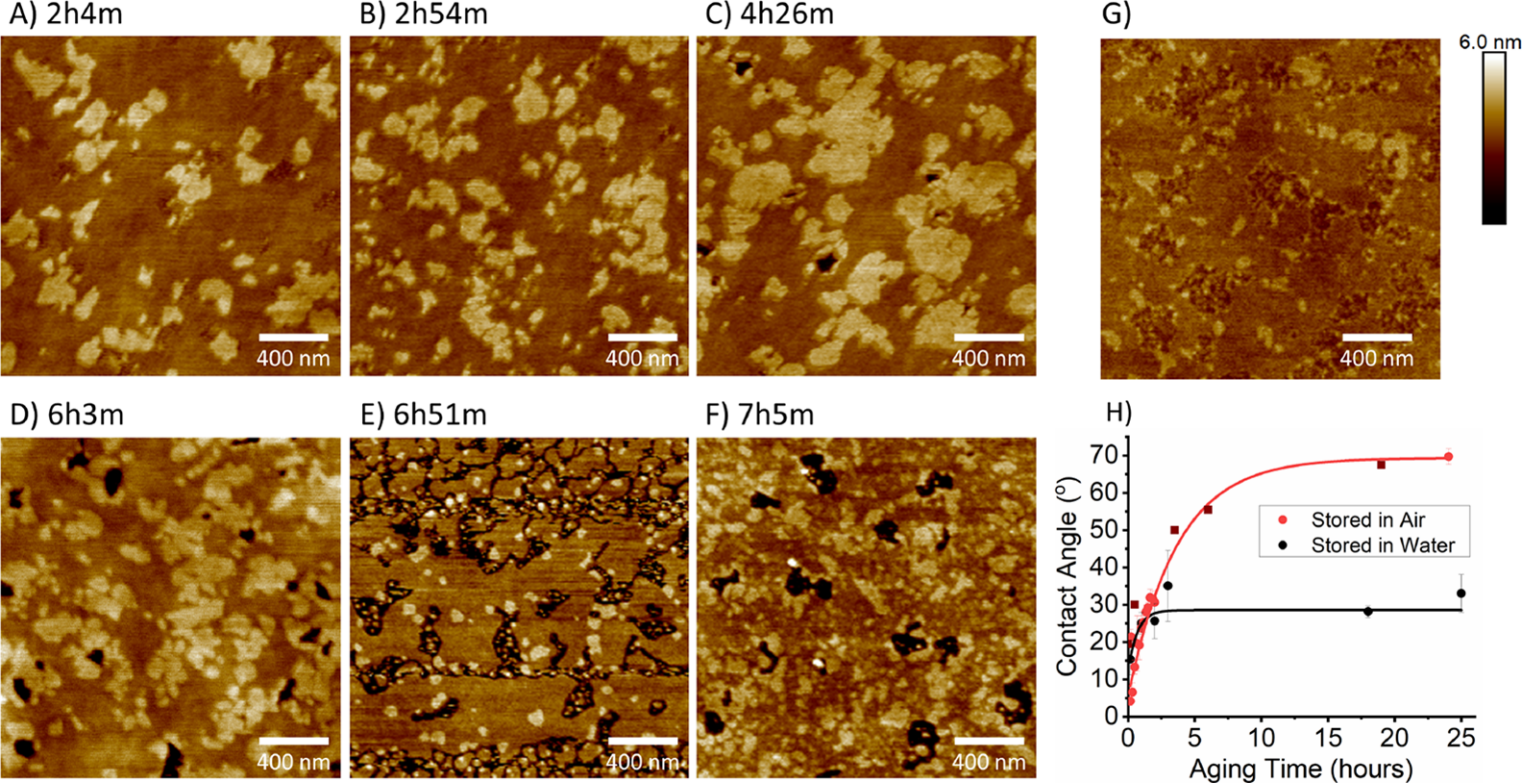
(A–F) AFM images of DPPC/DOPC (60:40) bilayers on plasma-oxidized PDMS, showing nanoscale domains. The time stamp refers to the time since PDMS oxygen plasma treatment. Each subsequent time-stamped image was taken on a different area of the bilayer. The images show defects appearing over time as the bilayer becomes less mechanically stable. (G) Another AFM example of a disrupted bilayer morphology on PDMS. (H) Contact angle recovery of oxidized PDMS over time when stored in air and in water as a proxy for a hydrated bilayer experiment. Hydrophobic recovery of PDMS in air is faster than in water. Each experimental point is a separate piece of PDMS stored in water for the specified time. Dark red squares are data taken from the original paper that explored hydrophobic recovery of PDMS, showing excellent correspondence to our data.^45^

It is well documented than PDMS undergoes hydrophobic recovery over time,^43−47^ with the paper by Jahangiri et al. providing a recent comprehensive overview of the literature.^47^ Hydrophobic forces are largely responsible for the self-assembly of lipids into bilayers, so it is no surprise that a bilayer on PDMS will be affected by the hydrophilic–hydrophobic balance of the substrate within nanometer proximity. The gel–liquid phase-separating mixture was deposited on freshly hydrophilized PDMS and displayed the nano-domain structure as described above. However, after approximately 3 h, the bilayer starts to progressively degrade (Figure 5; 2.0 μm AFM images), with pinhole defects appearing and then expanding. The phase structure is also affected. Conversely, a bilayer on mica or glass under liquid is stable for at least 2 days. Hovis et al. reported seeing patches of bilayers separating from PDMS SLBs on the hour/day timescale.^12^ Faysal et al. also observe large bilayer defects appearing by around 36 h on PDMS using fluorescence but not on glass.^30^ AFM resolution enables detection of bilayer damage forming at much earlier time points than optical microscopy.

**Figure 6:**
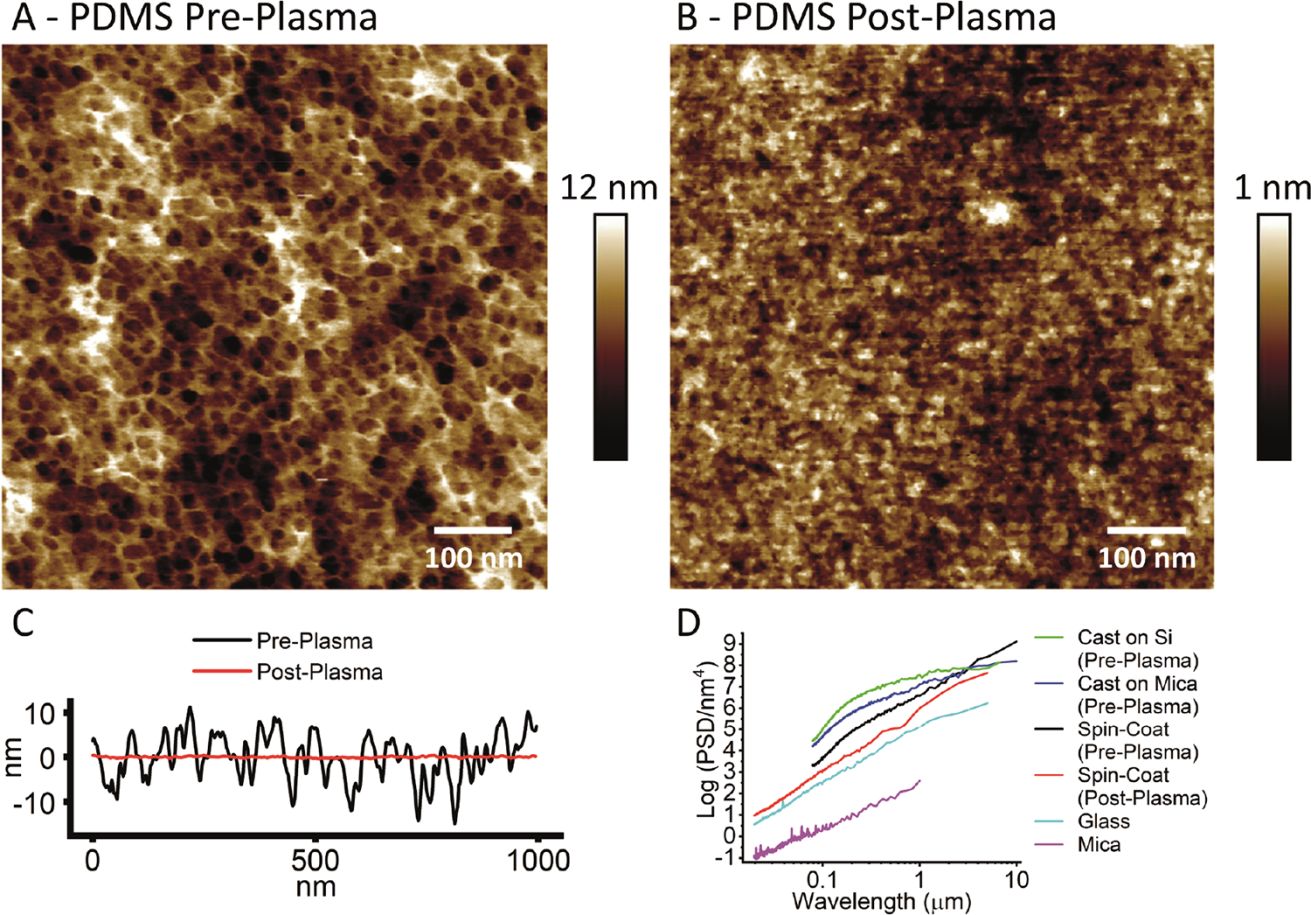
PDMS structure and roughness. (A) AFM image of spin-coated PDMS pre-oxygen plasma, *R*_a_ = 2.6 ± 0.9 nm. (B) AFM image of spin-coated PDMS post oxygen plasma, *R*_a_ = 0.21 ± 0.03 nm (n.b., the height scale is much reduced). (C) Height-section line scans across spin-coated PDMS pre- and post-plasma. (D) Power spectral density (PSD) plotted against the wavelength to quantify the relative roughness values over different length scales. PSD measured for spin-coated PDMS (pre- and post-oxygen plasma) as well as for PDMS prepared by casting against mica and silicon pre-oxygen plasma in order to control flatness. The PSD of mica is measured after cleavage, where the mica will be atomically smooth. This measurement reveals the noise floor of the AFM (30 pm). The PSD of glass is measured following piranha and UV ozone cleaning.

PDMS hydrophobic recovery is due to free unreacted siloxane monomers or low-molar mass oligomers in the bulk PDMS diffusing to the PDMS–air interface through the porous silica structure (clearly resolved using high-resolution AFM; Figure 6A), resulting in the gradual replacement of hydrophilic silanol (Si-OH) groups by hydrophobic methyl groups (Si-CH_3_).^43,44,46,48^ A secondary mechanism has recently been proven, where low-molecular weight siloxanes that have evaporated into the local environment have re-adsorbed onto the surface.^49^ This agrees with our experience where any PDMS materials and operations must be kept isolated from other surface-sensitive sample preparation and measurements due to contamination with hydrophobic siloxanes as they will coat every surface in the vicinity.

Hydrophobic recovery can be monitored using contact angle measurements (Figure 5H). Untreated PDMS is hydrophobic with a contact angle of 105 ± 2°, but after oxygen plasma treatment, the contact angle can be reduced to near 0° (although the final angle depends on the degree of treatment), similar to hydrophilic mica (3 ± 2°) or plasma-treated glass (5 ± 2°).^25^ To understand the degree of hydrophobicity driving bilayer degradation, the contact angle of PDMS stored in water was measured as a function of time. Although not a novel experiment in itself (for example, Zhao et al.^50^ studied hydrophobic recovery in air, pure water, and LB-broth, finding extremely variable results depending on multiple experimental parameters, and advanced contact angle measurements were used more recently by Wong et al.^51^ to understand some of this variability), this wide range of values calls for an experiment to understand the PDMS properties in our experimental setup, where it is unlikely that the plasma protocol we have optimized will have identical outcomes to the work of other labs.

Batches of PDMS were plasma-treated and placed in water (MilliQ) immediately. The first time point was measured without water storage, but all subsequent contact angles were measured on a PDMS sample taken out of the water sequentially (Figure 5H). At 4 h, when bilayer breakdown is starting to become obvious, the contact angle has recovered to above 30°. Fluid bilayers have been previously reported to form up to approximately 30°,^29,52^ but above this (around 60°), unruptured vesicles will absorb. In the range up to 100°, no vesicles absorb, and at >110° (i.e. untreated PDMS), a lipid monolayer will form.^30^

The hydrophobic recovery of PDMS in water is significantly slower than in air, shown in our data (Figure 5H) and in the literature.^50,52^ The thermodynamic drive for monomer diffusion and recovery is significantly reduced when the hydrophilic-surface silanol groups are in contact with water. However, a hydrated SLB on treated PDMS will still only be viable for several hours. Although storage under water (equivalent to deposition of a bilayer in aqueous condition during an experiment) drastically slows hydrophobic recovery above 30°, this is unfortunately around the contact angle where bilayer breakdown occurs, so is not sufficient to allow long-term experiments or preparation and storage of PDMS for future experiments.

Many attempts to prevent hydrophobic recovery have been described in the literature. These include storage at −80 °C, effectively freezing the migration of PDMS oligomers^47^ (although this would not help in subsequent experiments in liquid water); thermal aging of the PDMS in an oven prior to use, which has been shown to slow recovery to the critical 30° contact angle from 4 h to 4 days;^44^ and by coating with PVA following plasma oxidation,^53^ providing a longer-lasting hydrophilic surface, although the resultant contact angle varied from 20 to 40° and much of the time around 30° which (again) is the critical value for bilayer stability. Another polymer, HEMA, has also been similarly used,^62^ producing a stable contact angle of 10–15°, although this method from 2006 does not seem to have been adopted.

The most promising method developed recently is washing the PDMS in solvent to extract monomers/oligomers of PDMS with toluene^51,54^ or a sequential 15 min sonication in pure acetone and iso-propanol.^30^ The PDMS thickness determines the time required for solvent washing; for 10s of nanometers of spin-coated PDMS, 10 min is sufficient, but for a slab of several millimeters in thickness (such as used in microfluidic devices), a soak for 24 h is required.^54^ These methods result in much enhanced bilayer stability, as determined by the detection of intact bilayers via fluorescence intensity (vs disruption or half intensity monolayers) and by FRAP-determined diffusion coefficients, which are unaffected up to 5 days.^30^ However, the precise evolution of contact angles using solvent washing has yet to be determined to our knowledge.

Lippert et al. claims that by incubating PDMS overnight with CaCl_2_, plasma treatment is not needed to form fluid bilayers (confirmed by FRAP and FCS).^55^ We speculate that in this method, a lipid monolayer is formed on the hydrophobic PDMS and then a long incubation of lipid SUVs in a Ca^2+^-containing buffer bridges the charge repulsion and allows a bilayer or layers to form on top of the monolayer. This is supported by their AFM force spectroscopy curve, which shows a much larger than expected breakthrough distance of about 12 nm.

### Nanoscale Surface Structure and Roughness of PDMS

To assess how PDMS may differ from other commonly used bilayer substrates and the potential impact this may have on bilayer structure and behavior, the surface structure and roughness were measured using AFM. High-resolution peak-force tapping AFM images of spin-coated, cured PDMS before plasma treatment reveal a honeycomb-like open network structure (Figure 6A; n.b. older and lower-resolution images of this structural detail from our lab can be found in the recent publication by Liamas et al.^54^ ), with pores of 14.4 nm mean diameter (S.D. = 3.7 nm, *N* = 100) (Figure S3) and depth of approximately 15 nm. This maximum apparent pore depth represents the distance the AFM probes can penetrate due to tip–sample convolution, a function of probe sharpness and aspect ratio. The lateral length scale of this network structure reflects the distance between the strong covalent cross-links, a property known as the mesh size. However, this is a surface and not a cross section of the bulk, and structure can often be altered close to a surface or interface.

Following oxygen plasma treatment, this network structure disappears and is replaced by a much smoother glass-like surface (Figure 6B). The surface roughness drops by an order of magnitude, from *R*_a_ = 2.6 ± 0.9 nm pre-treatment to *R*_a_ = 0.21 ± 0.03 nm post plasma, both measured over 1 μm^2^ images. A more informative method for comparing roughness between samples is to use power spectra, which show the power of different length scale fluctuations in the 3D topography of a surface, in other words, roughness at different length scales. The power spectra in Figure 6D clearly show that the post-oxygen plasma roughness (red) is approximately an order of magnitude lower across all length scales measured, 100 nm–5 μm, compared to untreated PDMS (black). When the PDMS surface is exposed to oxygen plasma, XPS studies show that the [SiO(CH_3_)_2_] structure is replaced by a SiO_*x*_ silica structure with increased cross-linking Si–O bonds and silanol groups (Si–OH).^43,48,56^ The disappearance of the honeycomb polymer structure and the reduced roughness are the topographical results of this chemical change in the polymer structure. This extreme degree of surface smoothing we always find disagrees with a recent study by Tsuzuki et al.,^46^ who report that the surface roughens then falls back to a similar roughness to the original PDMS after prolonged treatment, although images are not provided. The only explanation we can propose for this is that they use UV-generated ozone to hydrophillize the PDSM and not high-energy O_2_ plasma.

The surface structure of PDMS was compared to the commonly used bilayer substrates mica and glass (Figure S2).^25^Figure 6D shows the power spectra of the three surfaces, with PDMS (after plasma treatment) rougher than glass (after piranha and UV ozone treatment) and much rougher than mica (after cleavage) across all length scales. The *R*_a_ values, measured over 5 μm^2^ images, match the trend in power spectra, with PDMS (0.28 ± 0.03 nm) being an order of magnitude higher than mica (0.03 ± 0.03 nm) with the glass intermediate (0.15 ± 0.03 nm). The roughness values and power spectra reflect the surface immediately before bilayers are incubated on the substrates.

We hypothesized that the roughness of PDMS could be reduced by casting and curing against flat substrates and that this might reduce the hindering of domain growth. When PDMS was cast against Si and Mica, the PDMS was indeed flatter over large >3 μm length scales (Figure 6D), where micrometer-scale corrugations are visible in the spin-coated PDMS but not when cast against silicon or mica (Figure S1). However, this PDMS was rougher over the <3 μm scale, compared to the spin-coated PDMS. This shows that the limiting factor is the nanoscale polymer network structure of the PDMS itself even when cast against an atomically flat mica surface. In fact, casting against the flat surfaces seems to exacerbate the roughness of the polymer structure (perhaps by setting the PDMS in a more open polymer conformation).

## Discussion

### Substrate Roughness Is Correlated with Lipid Domain Size

Bilayer phase separation has been demonstrated to take place on PDMS SLBs but with a nanoscale size similar to that seen on glass but significantly smaller than domains in free floating vesicles and SLBs on mica (Figures 2–4).^25^ Lipid diffusion was similar on PDMS, mica, and glass, so molecular mobility cannot explain the difference in domain size and morphology. There was a small but significant drop in *T*_m_ of a couple of degrees on both PDMS and glass compared to mica and vesicles, but this is not large enough to account for differences in phase separation (a more in-depth discussion of how diffusion and *T*_m_ might relate to domain formation is included in a previous paper^25^). Another key finding is that the sizes of the domains on PDMS do not increase as the cooling rate is reduced (Figure 2 and Table S1). The same behavior is seen on glass, but on mica, reducing the cooling rate increases the size of domains.^24,57^ This shows that even though the lipids have more time to diffuse and flow to form larger domains at slower cooling rates, the surfaces of PDMS and glass are acting to hinder the formation of large-scale domains.

We have shown that the surface of plasma-treated PDMS (0.28 ± 0.03 nm) is rougher but of a similar order of magnitude compared to glass (0.15 ± 0.03 nm) and is significantly rougher than mica (0.03 ± 0.03 nm). Supported bilayers conform to the micrometer-scale PDMS surface corrugations, where the bilayer surfaces show similar waves and corrugations to the bare PDMS substrates (Figure 2A–F). Bilayers on mica and glass also follow the surface but appear flat on the micrometer scale, as the micrometer-scale topography is relatively flat (Figures 2G,H and 4D). In a previous paper, we showed evidence that it is the nanoscale roughness, not the larger scale surface structure, that causes domain sizes to drop on glass compared to mica.^25^ As the *R*_a_ is devoid of in-plane information on the spatial frequency of roughness, power spectra are used to quantify roughness at relevant length scales (Figure 6D). Previously, roughness as a function of spatial frequency was measured for several different bilayer substrates at the 20 nm length scale, including mica, glass, silicon, and quartz. It was found that domain size drops as roughness increases,^25^ with a roughness threshold above which domains cannot grow. PDMS fits this trend (Figure S4) as it lies at the end of the sequence as the roughest surface, and it also found to be similar to a glass surface in all trends or domain sizes, diffusion coefficients, and transition temperatures. This is strong evidence that the roughness is the cause of hindered domain growth.

Mica roughened on the nanoscale using HF, resulting in 1.0 nm steps, also hindered domain formation, suggesting that topography and not chemistry is the overriding factor.^25^ Attempts to form smoother PDMS at the nanoscale were not successful as the roughness at this scale is a function of the polymer cross-linking and porous network structure (Figure 6 and Figure S1).

### Substrate Roughness Affects Hydrodynamic Lipid Flow

In a previous publication, we discussed how domain formation on rough surfaces can be restricted due to friction and disruption of hydrodynamic lipid flow.^25^ Decades-old observations show that roughness can slow the spreading of lipids across a surface by 1–2 orders of magnitude.^58^ On atomically flat mica, once a gel domain has nucleated, it is able to grow to micrometer size via lipid diffusion, and then by hydrodynamic flow of collective bodies of lipids, leading to the coalescence of smaller domains into larger domains, a process called ripening. They can also grow further via Ostwald ripening, where lipid molecules diffuse from small domains and move to more energetically favorable large domains due to the lower domain perimeter per lipid molecule. On rougher substrates such as PDMS, however, the domains reach a critical size where they become pinned to the surface.

There are two possible mechanisms by which roughness can disrupt lipid flow. First, rough surfaces can provide local pinning sites where the substrate-to-bilayer distance is smaller and the friction is larger; in effect, the domain is ‘beached’ at a single point.^58^ Second, rougher surfaces and more highly curved areas mean that the bilayer has an energy penalty for bending to map to the surface.^59^ If this energy penalty is too high, then the more rigid solid domains will not be able to flow over this area. The two friction mechanisms between the surface and the bilayer will be mediated by the thin 0.3–2 nm lubricating interstitial water layer, which has been shown to affect the spreading of lipids.^34,59^ The roughness of the different surfaces could also affect the molecular arrangement, thickness, and viscosity of the interstitial water layer. It is also possible that surface chemistry is influencing domain formation, but the effect is weaker, and the very similar behavior of PDMS to glass, and the properties of HF roughened mica, would argue against this. Surface chemistry only seems to take effect when the forces of bilayer self-assembly are disrupted due to hydrophobic recovery once the contact angle exceeds 30°.

Stubbington et al. show that when bilayers are controllably stretched and compressed on partially hydrophilic PDMS (mildly treated with plasma, water contact angle = 35–60°), membrane sliding is prohibited.^60^ However, on hydrophilic PDMS that fully wets, membranes can slide up to 10% PDMS expansion. Above 10% expansion, pores open up and then reform once the PDMS relaxes to original size. The motion of the domain boundaries with expansion and contraction is anomalous, with some areas constant and other regions seeming to flow on a fine length scale. We have shown it is probable that the PDMS structure hinders bilayer hydrodynamic flow via pinning.

A similar hindering effect on lipid phase separation is also observed when a minimal actin cytoskeleton network is pinned to a phase-separating SLB.^61^ Domain sorting is observed on SiO with pores to mimic the cytoskeleton, where the sorting is explained by the adhesion/pinning and bending moduli.^15^ Our results show that the rough PDMS surface can act to pin domains and restrict their growth similarly to the cytoskeleton in vivo. Despite SLBs being altered from their equilibrium state and from the simpler biophysical models of GUVs, the supported systems may actually be more biologically accurate. We also then have the chance to build and design better cell membrane mimics by controlling the surface chemistry, roughness, porosity, etc. of the support to match the cytoskeleton.

### Re-Evaluation of Bilayer Formation on PDMS in Literature

Hydrophobic recovery or incomplete PDMS oxidation can potentially explain certain findings. For example, when fluid-phase lipid diffusion on PDMS is compared directly to glass in literature, it is shown to be faster,^12^ similar,^30^ and 50% slower.^23^ The latter study measures the PDMS contact angle at 30°, which we find to be on the threshold of bilayer disruption caused by hydrophobic recovery (Figure 5), which could explain the reduced diffusion. Similarly, the drop in diffusion of DPPC on PDMS compared to glass and mica (24%), which is not observed on DOPC, could be due to the longer time taken to heat DPPC above its solid-to-liquid transition temperature, during which time the PDMS hydrophobicity is recovering, resulting in small defects (undetectable below the diffraction limit), which acts as pinning sites and affect diffusion.

Despite there being thousands of papers on phase separation in SLBs and many groups working on bilayers on PDMS, forming domains spontaneously on PDMS has proven elusive, hence this paper. There have been no reports of domain formation directly from vesicle rupture despite this being widely reported on mica.^1,5,8,10,41^

Previous studies utilizing PDMS to study curvature in membranes used GUVs ruptured onto PDMS, where the domains are already present in the GUV before deposition and the phase structure is locked in.^24^ On glass, domains in surface-ruptured GUVs dissipate when heated about the thermal transition but do not re-form upon cooling,^63,64^ which we previously attributed to roughness^25^—we expect the same thing to happen to ruptured GUV domains on PDMS. Another study used PDMS to investigate how phase-separated domains align on curved substrates, but they use a double bilayer (a challenging technique to control), which decouples the bilayer from the substrate.^17^ When bilayers are formed directly onto PDMS using SUV vesicle rupture, large-scale domains do not form, and our data explains why the decoupling or rupture of pre-existing domains was necessary for phase-separation experiments.

The apparent lack of lipid phase separation on PDMS has been noted in the literature, attributed to strong coupling with the substrate. This reflects similar observations of static bilayers on glass created from Langmuir–Blodgett transferred monolayers, where phase separation was only seen if it pre-existed in the monolayer, and the domains in the leaflets would not register.^63^ The only work showing visible domains directly on PDMS is by the Parikh group, using a phase-separating mixture deposited on a pre-strained PDMS substrate.^31,65^ Upon release, the PDMS returns to its original size, resulting in the incompressible oxidized glassy surface forming wrinkles. Before wrinkling, there are no domains observed optically, in agreement with our findings. The explanation given is that local curvature causes a dynamic domain reorganization, leading to domain formation. The domains are characterized by an absence of fluorophore, but the domain size and morphologies observed do not match any previously observed (on which the authors comment) or any domains we have observed on PDMS in this study and also did not recover during FRAP (hence being immobile). Conversely, a bilayer deposited on the pre-wrinkled surface (i.e., exhibiting curvature) again did not show domain structure but did recover during FRAP. This could possibly be explained by strong substrate coupling forcing local bilayer compression, ordering, and expulsion of the fluorophore, appearing as “domains”.

## Conclusions

In summary, PDMS can be used for model bilayer systems, but it is not possible to create bilayers that phase-separate at optical length scales due to the surface roughness hindering the collective hydrodynamic flow of lipids. Despite the surface oxidation procedure resulting in the transformation of the porous polymer network into a smooth glassy surface orders of magnitude smoother, the resultant surface is still too rough at the nanoscale, similar to but slightly rougher than glass. The intrinsic structure of PDMS will always have a limiting roughness, even when cast against atomically flat mica, and this will have ramifications for anyone trying to use PDMS as a flexible or nanostructured substrate.

On the positive side, lipid bilayers show free diffusion and phase-separated domains do exist at the nanoscale (<100 nm), which could be viewed as a more realistic model of phase separation as thought to exist in cells. DPPC exhibits a phase transition while supported on PDMS with only a small decrease in *T*_m_ compared to free-floating GMVs and mica but similar to glass, which can be explained by the small disordering effect of the surface roughness.

Care must be taken to ensure that the surface is fully oxidized and fully hydrophilic and that the time limit before hydrophobic recovery reaches a critical threshold of 30° water contact angle is considered; otherwise, the bilayer properties can change. Incomplete surface oxidation or hydrophobic recovery of PDMS can result in membrane defects, restricted lipid flow, reduced lipid diffusion, and extraction of small membrane components. Many methods exist to address the hydrophobic recovery of PDMS,^30,44,47,51−54,63^ and the most suitable should be chosen depending on experimental needs. PDMS-supported bilayers have already found multiple applications as PDMS can be rapidly fabricated into a wide array of architectures and can be stretched and compressed, for example, in the study of proteins on curved surfaces,^16−19^ localization of proteins in microcavities,^20,21^ and for mimicking the mechanical stress of cells.^60^ We hope that this detailed understanding of how PDMS affects supported bilayers will aid the interpretation of results and development of more effective strategies to utilize PDMS-supported bilayers. PDMS-supported SLBs will surely have further applications in lab-on-a-chip and microfluidic applications such as for high-throughput immunoassays^22,23^ as well as for new microfluidic applications such as organ-on-a-chip. Finally, we believe that PDMS may enable the building of more accurate membrane models that can replicate the roughness, geometries, porosity, and elasticity of the cytoskeleton.

## Data Availability

The data associated with this paper are openly available from the University of Leeds Data Repository at https://doi.org/10.5518/1372.

## Supporting Information Available

(Figure S1) AFM images of PDMS cast against mica helping show improvement in large-scale flatness but increase of nanoscale roughness of the network structure; (Figure S2) AFM images and line scans of clean mica, glass, and PDMS before bilayer deposition; (Figure S3) analysis of PDMS pore diameter measurements from high-resolution AFM images; (Table S1) summary of roughness and bilayer domain size measurements (using both particle analysis software and correlation length analysis) on PDMS, mica, and glass; (Figure S4) graph of the relationship between surface roughness and the size of lipid domains between different substrates; (Figure S5) example of the nanodomain size analysis using correlation curves; (Figure S6) FRAP recovery curves for pure DOPC on O_2_ plasma-treated PDMS (PDF)
