# Supplementary material for: PDMS as a Substrate for Lipid Bilayers
Source: Langmuir. 2023 Jul 26;39(31):10843–54. doi: 10.1021/acs.langmuir.3c00944 (PMC10413950; doi:10.1021/acs.langmuir.3c00944)
Supplement: Supplementary file 1 — la3c00944_si_001.pdf [file la3c00944_si_001.pdf]

PDMS as a Substrate for Lipid Bilayers –

SUPPORTING INFORMATION

James A. Goodchild[a§], Danielle L. Walsh[a§], Harrison Laurent[a], Simon D. Connell[ab*]

[a] Molecular and Nanoscale Physics Group, School of Physics and Astronomy, University of Leeds, Leeds LS29JT, United Kingdom.

[b] Bragg Centre for Materials Research, William Henry Bragg Building, University of Leeds, Leeds LS2 9JT, United Kingdom.

*E-mail: s.d.a.connell@leeds.ac.uk

Figure S1

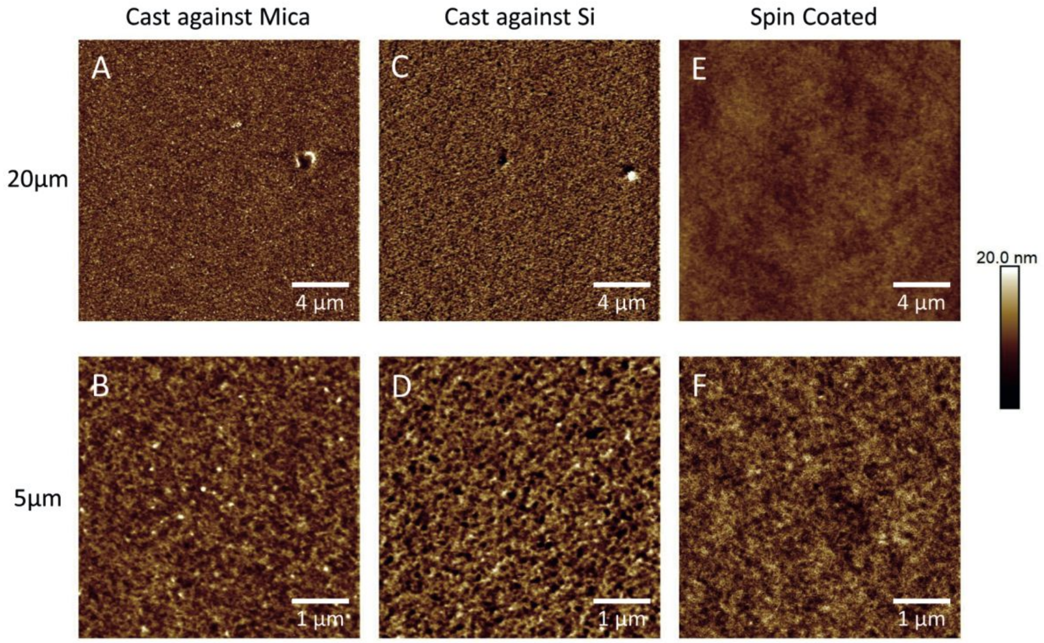

Figure S1. AFM images of PDMS structure. A)+B) PDMS Cast against Mica. C)+D) PDMS cast against Silicon. E)+F) Spin-coated PDMS. All PDMS samples are pre-plasma treatment. A),C) and E) are 20 μm² images. B), D) and F) are 5 μm² images. The roughness of the different samples is represented as power spectra in Fig. 6D, where it can be seen that over large length scales (3 μm+) the PDMS cast against Si and Mica is flatter, but at lower length scales (100 nm - 3 μm) the surface is actually rougher. R_a roughness values of 5 μm² images are 1.53±0.03 nm (cast against mica), 2.3±0.3 nm (cast against Si), and 1.3±0.3 nm (spin-coated).

Figure S2

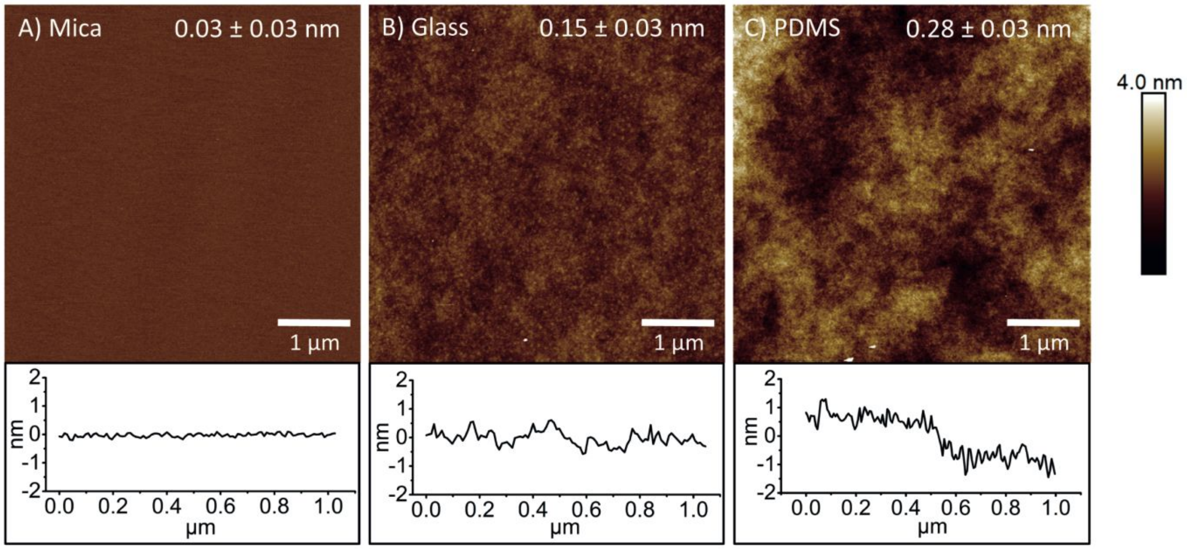

Figure S2. AFM images of bare substrates with no bilayers. A) Mica after cleavage. B) Glass after Piranha and UV Ozone clean. C) PDMS after oxygen plasma. R_a roughness values are quoted in the top corner of each image and are averages with standard errors across multiple 5 µm^2 images. For reference, the PDMS roughness values in Fig. 6 are measured over 1 µm^2 images. Single line scans of 1 µm lines are shown below each image.

Figure S3

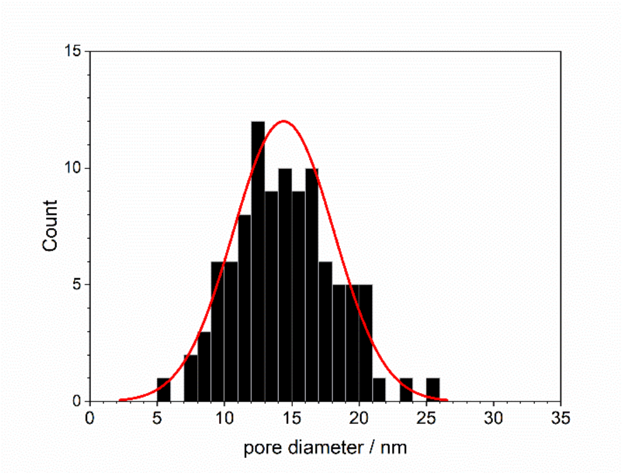

Figure S3. Histogram of PDMS pore size measurements from Fig. 6A, with gaussian fit.

Table S1

Substrate	Domain Radius (AFM) / µm	Correlation Length (AFM) / µm	Substrate Roughness (R_a) / nm
**PDMS**	Analysis fails	0.049±0.007 µm	0.28±0.03
**PDMS (slow cool)**	Analysis fails	0.037±0.008 µm	0.28±0.03
**Mica**	5.3±0.2	2.3±0.4 µm	0.03±0.03
**Mica (slow cool)**	8.2±0.8 µm	3.3±0.1 µm	0.03±0.03
**Glass**	Analysis fails	0.074±0.005 µm	0.15±0.03
**Glass (slow cool)**	Analysis fails	0.065±0.007 µm	0.15±0.03

Table S1. Domain Sizes and Correlation Lengths for DPPC/DOPC(60:40) domains on PDMS, mica and glass. Cooling rates from incubation temperature down to room temperature are all ambient (0.25±0.02 °C/min) except where specified as slow cool (0.080±0.008 °C/min). Domain Fitting was not possible on nanoscale domains due to irregular and interconnected morphology. R_a roughness values are measured over 5 µm^2 images. For reference the R_a roughness values in Fig. 6 are measured over 1 µm^2 images. 0.03 nm is the noise floor of the AFM, mica will actually be atomically smooth.

Figure S4

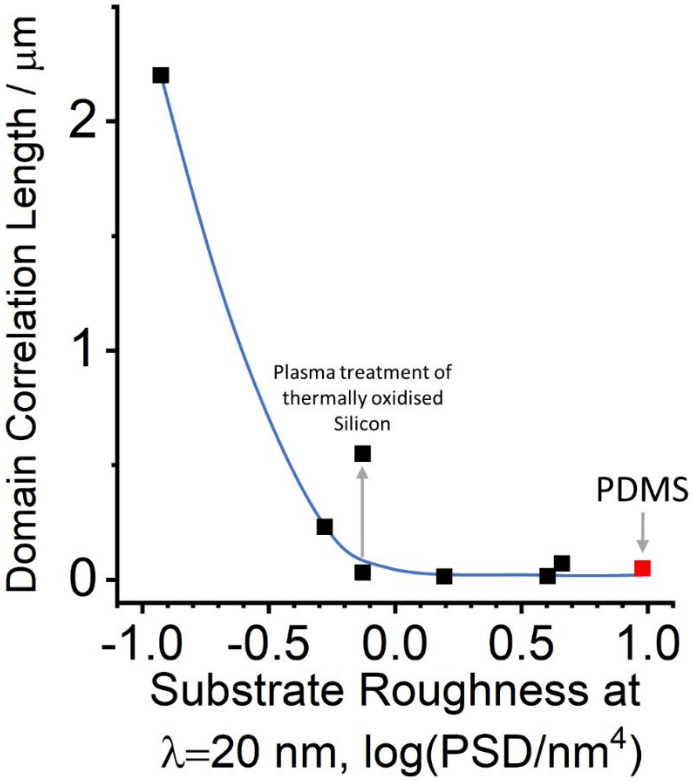

***Figure S4. Graph showing the correlation length of domains with power spectral density (PSD) roughness of surfaces. This figure is replicated from our previous paper,*[25] *with the PDMS data point added. The blue line is a guide to the eye. Domains formed on thermally oxidised silicon vary in size dependent on plasma treatment, suggesting its roughness is around the level where surface chemistry can switch behaviour over the threshold between forming large and small domains. This is discussed in more detail in our previous publication.*[25]**

Figure S5

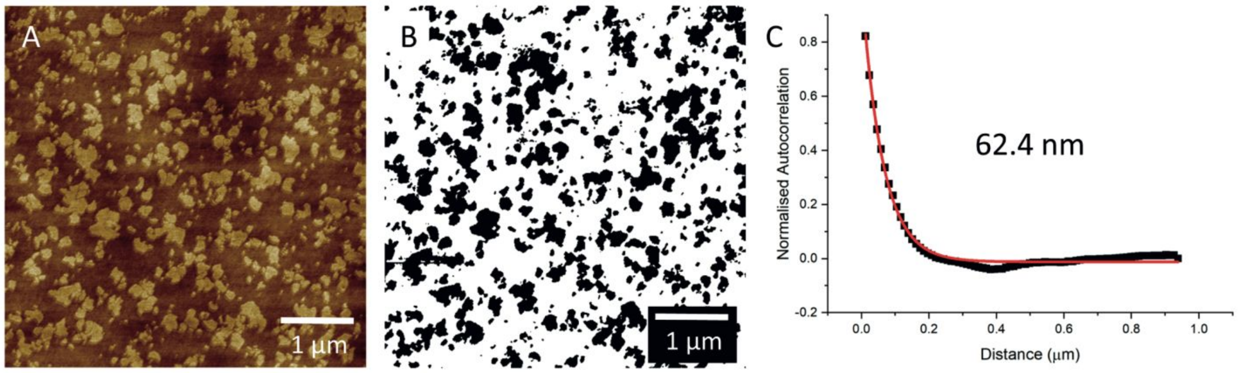

Figure S5. Example correlation length analysis for domains on PDMS formed with ambient cooling. A) AFM image showing gel and liquid phase coexistence on PDMS. B) Binary Image of two phases. C) Autocorrelation curve with exponential decay fit to calculate correlation length (correlation length displayed on graph).

Figure S6

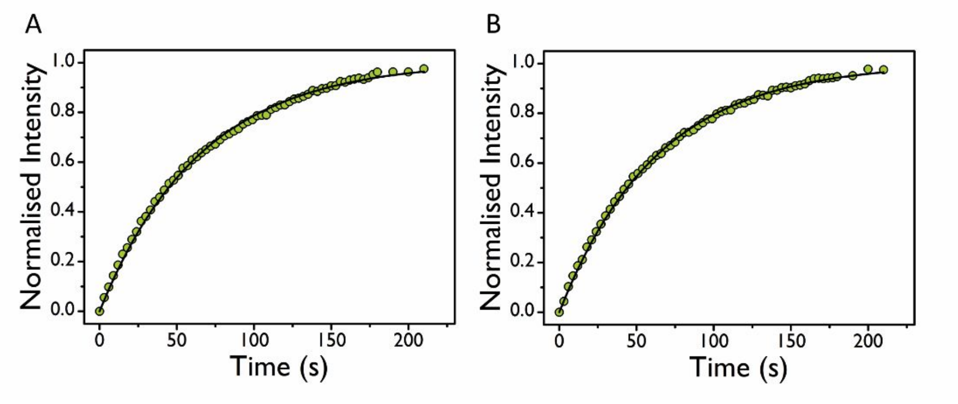

Figure S6. Example FRAP Recovery curves (normalised intensity vs time) for DOPC + 0.5 mol % TR-DHPE on O_2-plasma treated PDMS.
